# Freeform Mode-Engineered Metasurfaces

**DOI:** 10.1021/acs.nanolett.5c06075

**Published:** 2026-03-10

**Authors:** Zhongjun Jiang, Tianxiang Dai, Shuwei Guo, Soyaib H. Sohag, Yixuan Shao, Chenkai Mao, Andrea Alù, Jonathan A. Fan, You Zhou

**Affiliations:** † Department of Physics and Optical Science, University of North Carolina, Charlotte, North Carolina 28223, United States; ‡ Department of Electrical Engineering, 6429 Stanford University, Stanford, California 94305, United States; § Photonics Initiative, Advanced Science Research Center, City University of New York, New York, New York 10031, United States; ∥ Physics Program, Graduate Center, City University of New York, New York, New York 10016, United States

**Keywords:** nonlocal metasurfaces, topology optimization, high-*Q* nanophotonics, chirality, Mie resonance

## Abstract

Nanophotonic technologies inherently rely on tailoring light–matter interactions through the excitation and interference of deeply confined optical resonances. However, existing concepts in optical mode engineering remain heuristic and are challenging to extend toward complex and multifunctional resonant phenomena. We introduce an inverse design framework that optimizes near-field distributions, ideally suited to tailoring Mie-type modes within dielectric nanophotonic structures, and we demonstrate its application to the discovery of new classes of nonlocal metasurfaces. We show that freeform nonlocal metasurfaces supporting accidental bound states in the continuum can be readily optimized for tailored illumination conditions, modal properties, and quality factors. We further generalize the framework to higher-order and multifunctional mode engineering and experimentally demonstrate freeform planar nonlocal multiwavelength and chiral metasurfaces. Our versatile framework for freeform mode engineering has applications in broad high-quality-factor nanophotonic platforms relevant to sensing, nonlinear optics, optomechanics, and quantum information processing.

## Graphical abstract

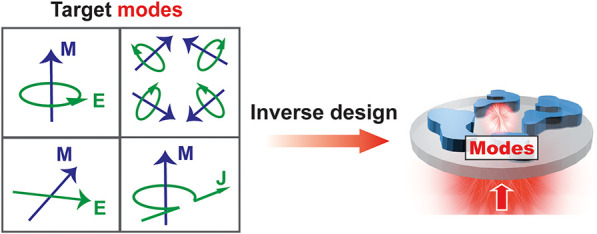

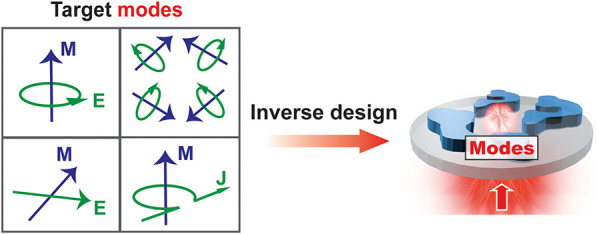

Light–matter interactions bridging free-space waves and nanoscale resonant modes are crucial in the quest to engineer near- and far-field optical responses in nanophotonic technologies. Modal descriptions of photonic media provide a basis for delineating and engineering near-field hotspots essential for enhancing molecular sensing,^1–3^ fluorescence emission,^4–7^ and optical nonlinearities.^8–11^ In the far field, designed optical antennas with customized modes support tailored scattering profiles and can collectively function as optical phased arrays.^12,13^ Using the more general framework of Mie resonance engineering, the tailored excitation, coupling, and interference of electric and magnetic multipolar resonances serve as the basis for the Kerker effect^14^ and tailored bianisotropy,^15–18^ which are utilized in Huygens’ metasurfaces,^19–21^ optical cloaks,^22–24^ and large-angle metagratings.^15,25–27^ Recent research efforts have applied these ideas to engineered nonlocalities, based on guided mode resonances and quasi-bound states in the continuum (BIC),^28,29^ which can be tailored to enable far-field spectral filtering^30–32^ and wavefront engineering with narrow band responses.^33–36^

Despite the important role of resonances in this quest, a pathway to rationally tailor customized optical modes in structured media remains elusive due to the lack of precise analytical correlations between nanoscale geometry and near-field distributions. This observation is emblematic in the typical design process of nonlocal metasurfaces,^37^ which consists of a combination of physical intuition combined with numerical experiments. In a typical workflow, nanostructure geometries featuring nonradiating optical modes are proposed and identified using known physical relationships between nanostructures and modal symmetries. The layout symmetry is then carefully broken with spatially tailored perturbations to enable weak coupling pathways between these highly confined modes and free-space radiation. Full-wave simulation sweeps are critical to empirically relating symmetry breaking with nonlocal responses and building an alphabet of perturbations that allows the spatial structuring of nonlocal modes.^36^ These approaches have been highly effective at developing the foundation of nonlocal metasurface research.^1,9,38–40^ However, the complexity of local mode engineering and nonlocal responses that can be realized with these methods is limited, and it is challenging to extend these concepts to nonintuitive geometric shapes that support full customization of multiple modes and functionalities within a single metasurface platform. It is not even clear whether there are fundamental limits to how many modal responses and functionalities can be packed within a single ultrathin metasurface. In addition, the role of symmetries in the initial design makes it easier to tackle radiation toward high-symmetry points, and sophisticated dispersion engineering needs to be explored to rationally design metasurfaces with lower symmetry radiation.^41^

In the following, we introduce a computational framework for freeform optimization of Mie-resonant metasurfaces, based on adjoint optimization,^42,43^ which enables the explicit design of customized optical modes in the near-field. While inverse design techniques have been widely used to shape the far-field wavefront responses of metasurfaces,^25,44–49^ the design of physical nanostructures that support customized modal responses has remained underexplored. Our approach bridges nanoscale mode engineering with freeform topology optimization, enabling the optimization of high-quality-factor (*Q*-factor) metasurfaces within an exceptionally large design space and facilitating the discovery of new classes of nonlocal metasurfaces with a complex nanophotonic response. The workflow of our computational approach is presented in Figure 1. Given a desired near-field or far-field metasurface response, we first build a framework of the desired optical mode physics (Figure 1, left) and specify the mode profile, orientation, *Q*-factor, wavelength, and complex amplitude. To tailor the coupling between free-space waves and the desired nanoscale modes, we utilize an adjoint variables method (AVM)^42,43^ adapted to the near-field, in which forward and adjoint simulations utilize a combination of near-field and far-field excitation sources (Figure 1, right). Our study uses a basic local gradient descent optimizer, though the local gradients calculated using AVM can ultimately be used in conjunction with a wide range of local and global optimization algorithms.^27,49,50^ For this study, we use far-field signatures as a straightforward, experimentally accessible readout mechanism of the engineered modal states, which relate via coupled mode theory^51,52^ (Supplementary Section 1).

**Figure 1:**
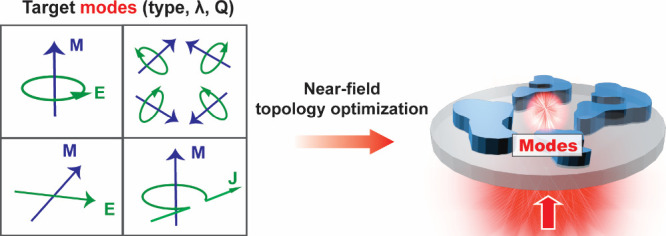
Mode engineering framework. The process starts with identification of optical modes as design objectives (left), and their mode profiles, *Q*-factors, wavelengths and complex amplitudes are optimized through near-field topology optimization (right).

Our freeform optimization strategy, in which low-performing geometric layouts evolve toward high-*Q*-factor structures with desired modal profiles, supports distinctive features compared to conventional nonlocal metasurface design approaches. Our platform is ideally suited for full-wave solvers and fully accounts for and exploits the complex relationship between nanoscale freeform shape and optical mode properties without approximations. It does not need to assume or enforce high symmetries pertaining to the photonic nanostructures and incident waves, and it is therefore particularly useful at discovering new classes of accidental BIC structures that are challenging to identify through heuristic designs. Our platform also readily extends to devices hosting multiple multipolar resonances using multiobjective optimization, and it can fully tailor the wavelength, *Q*-factor, complex amplitude, and spatial position of each mode in the device.

To illustrate the basic concept with a simple model system, we consider the design of a nonlocal, periodic silicon metasurface as a numerical testbed for mode engineering. We select an operating wavelength of 1500 nm with a period of 750 nm along the *x*- and *y*-directions, and we tailor the device to couple a normally incident plane wave to an out-of-plane magnetic dipole Mie mode (*m*_*z*_) (Figure 2a). The *m*_*z*_ mode has been identified in prior quasi-BIC-based metasurface demonstrations as the basis for high-*Q* metasurface implementation.^31,34,37,38,53^ To ensure that only this Mie-type mode is supported in the metasurface, the film thickness is limited to 150 nm (≈ λ/10), suppressing higher-order multipolar resonances. Furthermore, the planar metasurface unit cell boundaries are specified to be air to isolate the meta-atom structures from their neighbors, which suppresses the formation of delocalized mode profiles spanning multiple unit cells (see details in Supplementary Section 2).

**Figure 2:**
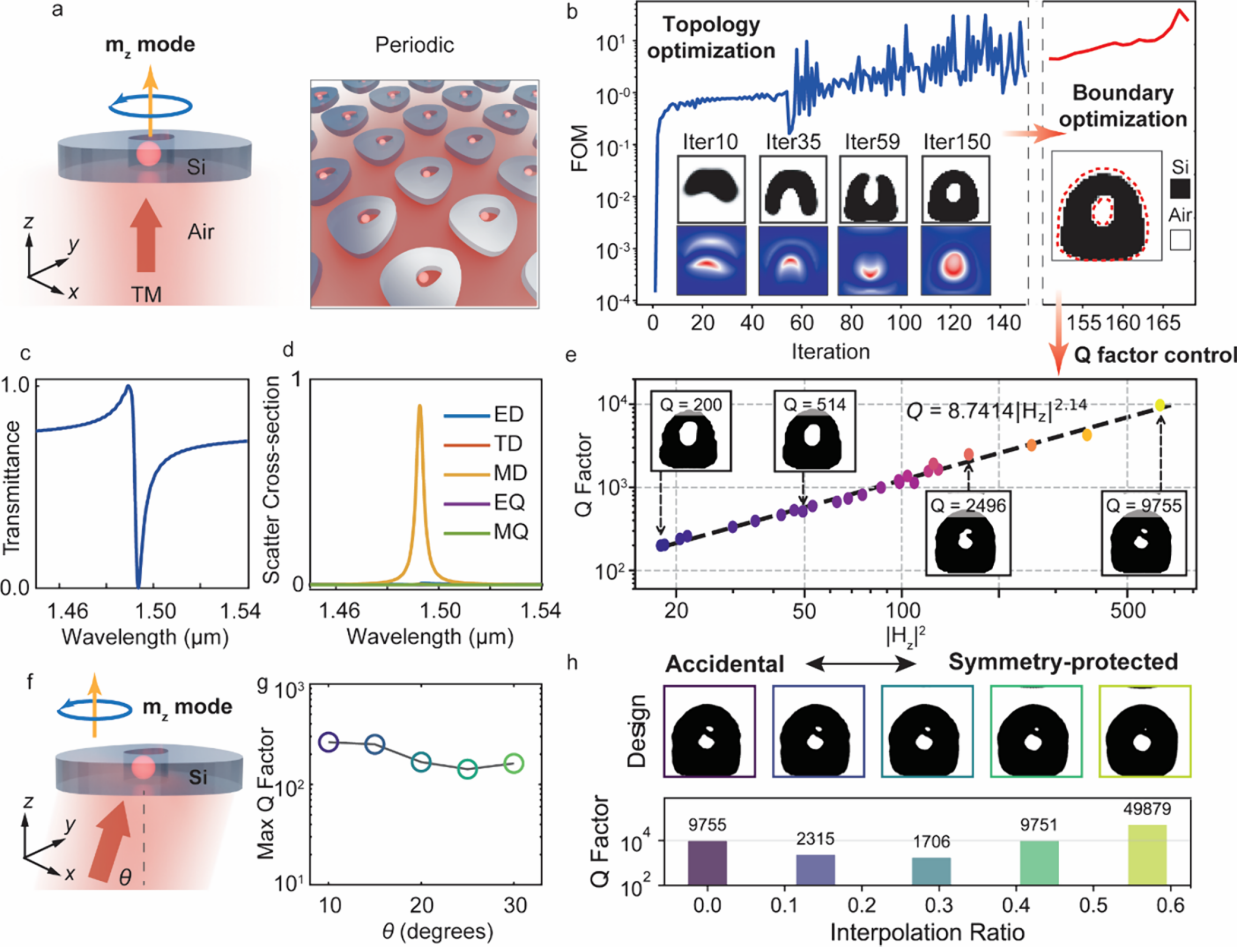
Freeform nonlocal quasi-BIC metasurfaces. (a) Left: schematic of a nonlocal metasurface supporting a quasi-BIC mode featuring a vertical magnetic dipole (*m*_*z*_) Mie mode. Right: a bird’s-eye view of the periodic metasurface that supports magnetic hotspots. (b) Optimization trajectory showing the enhancement of the FoM over the course of topology and boundary optimization. Insets: structural and magnetic near-field evolution within a single unit cell. (c, d) Transmission spectrum (c) and multipolar decomposition (d) of the optimized metasurface after the topology optimization stage. Abbreviations: ED (electric dipole), TD (toroidal dipole), MD (magnetic dipole), EQ (electric quadrupole), MQ (magnetic quadrupole). (e) *Q*-factor engineering using neuro-parametrized boundary optimization. Insets: unit cell structures for different target *Q*-factor values. (f) Schematic of a nonlocal metasurface supporting accidental BICs under oblique incidence. (g) *Q*-factors of optimized metasurfaces designed for different off-axis incident angles. (h) Shape interpolation of the device supporting an accidental BIC mode to a symmetric donut supporting a symmetry-protected BIC mode. Top: geometric evolution of the unit cell with increasing interpolation ratio. Bottom: *Q*-factor as a function of interpolation ratio, showing a nonmonotonic transition between the two BIC regimes. The unit-cell period is 750 nm for all designs.

A figure of merit (FoM)^31,32^ is defined to maximize the complex amplitude of the desired *m*_*z*_ Mie fields in the metasurface, given the desired incident far-field excitation source. A precise AVM setup for our problem therefore involves the forward source being a normally incident wave and the near-field adjoint source capturing the *m*_*z*_ Mie mode profile. Given the finite spatial extent of the modal profiles, the natural FoM for this problem would be to maximize a spatial overlap integral with the target near-field distributions.^54,55^ Interestingly, we have found that it is possible to simplify our FoM to the maximization of the complex field amplitude at a point centered within the silicon metasurface unit cell and to use the excitation of a point dipole *m*_*z*_ source when performing adjoint simulations. Our use of a point dipole adjoint source yields a simple and numerically stable FoM and is effective because our nonlocal metasurface system exhibits an enhanced Mie mode optical density of states, and as the photonic Mie mode dielectric structures form during optimization, coupling between the point source and emergent Mie structure leads to predominantly Mie mode-based near-field profiles.

Our optimization algorithm utilizes a two-part AVM-based optimization pipeline (see detailed workflow in Supplementary Section 3). First, density-based topology optimization is performed to identify metasurface topologies that roughly capture the desired coupling between the far-field source and near-field modes. Second, we fine-tune the modal properties and *Q*-factors using AVM-based boundary optimization. A challenge posed by the optimization of high *Q*-factor photonic devices is the extreme sensitivity of the device properties to geometric perturbations. To address this challenge, we introduce a neuro-parametrization scheme to describe the metasurface layout, in which a neural network encodes analytic relationships between position and device layout.^56^ Such a scheme circumvents spatial resolution limits posed by density-based AVM design concepts by specifying layout features with unlimited spatial resolution. It also introduces new ways to include constraints important to experimental fabrication, such as feature size and curvature constraints, by framing constraints in the form of loss function engineering during network training and geometry updating.

The optimization trajectory tracking the FoM is presented in Figure 2b and shows three parts. First, the design is initialized as a uniform grayscale permittivity profile, and a density-based AVM optimization is performed with continuous grayscale dielectric values to identify a promising device topology. Over the course of optimization, the FoM consistently increases and is ultimately enhanced by five-orders-of-magnitude compared to the starting FoM (Figure 2b, blue line). Second, the device evolves into a binarized freeform structure consisting of Si and air, and the magnetic field distribution within the structured media gradually transforms into a localized hot spot, indicating the emergence of a high-*Q* magnetic resonance (Figure 2b, inset). The transmission spectrum of the metasurface after this stage (Figure 2c) features a narrow-band Fano resonance dip within a broadband transmission window, which is typical of the interference between radiative and nonradiative modes. To confirm the excitation of the *m*_*z*_ Mie mode in the metasurface, we performed a multipolar decomposition of the metasurface near-fields from the current density distributions induced in the nanoscale resonators.^57^ As shown in Figure 2d, the modal decomposition reveals the excitation of a dominant magnetic dipole resonance and no additional noticeable modes. Third, the FoM is fine-tuned using AVM-based boundary optimization with our neuro-parametrization scheme (Figure 2b, orange line) to further push the *Q*-factor limit. The detailed tuning of the *Q*-factor as a function of the out-of-plane magnetic field intensity *|H*_*z*_ *|* ^2^ is shown in Figure 2e and shows a linear trend consistent with the relation FE^2^ ∝ *Q*, where FE denotes the local electromagnetic field. This trend is consistent with those known for critically coupled, lossless single-mode systems.^58^ The insets show the gradual geometric modifications of the high-resolution features, leading to varying *Q*-factors, with the highest value reaching 10^4^.

The designed meta-atom exhibits an asymmetric “donut” shape that resembles previously studied symmetry-protected designs;^37,59,60^ however, our structure ultimately utilizes distinct physics. Symmetry-protected BICs require symmetry constraints pertaining to the photonic nanostructures and the incident wave, typically emerge at the center of the Brillouin zone, and are supported in highly symmetric arrays operating under normal incidence. In contrast, our approach facilitates accidental BIC formation purely from structural engineering,^61,62^ enabling new classes of nonlocal metasurfaces featuring asymmetric geometries and illumination conditions. As a demonstration, we designed a series of accidental BIC metasurfaces for different oblique incidence angles (Figure 2f). As shown in Figure 2g, the *Q*-factors of the optimized metasurfaces may be consistently pushed above high values with minimal shift in the resonance frequency (Supplementary Section 4). To confirm that the freeform-designed BICs are accidental and not symmetry protected, for the device optimized for normal incidence, we perform a shape interpolation between the freeform structure and that of a symmetric donut structure. As shown in Figure 2h, the corresponding *Q*-factors decrease during the intermediate stages of shape interpolation and then increase again as the geometry approaches the symmetric layout. This nonmonotonic trend highlights a transition between the accidental and symmetry-protected BIC schemes (see detailed field analyses and additional freeform accidental BIC designs in Supplementary Sections 5 and 6).

Our approach can be generalized to the specification of higher-order Mie modes (Figure 3a) by defining more complex adjoint sources tailored to the corresponding near-field modal profiles. To demonstrate, we optimize freeform metasurfaces that host clean electric and magnetic quadrupoles, thereby providing a route toward the coupling of far-field radiation to dipole-forbidden processes relevant to nonlinear optics,^63^ surface-enhanced Raman scattering,^64,65^ and quantum emission enhancement.^66,67^ A schematic design setup for the electric quadrupole (EQ) mode (Figure 3b) shows the adjoint sources are implemented by placing four in-plane oriented electric dipoles inside the unit cell, positioned and oriented to match the desired quadrupolar field distribution.^68,69^ We employ a multiobjective FoM defined as the sum of pointwise field intensities at these four probe locations. The multipolar decomposition (Figure 3b, right) of the designed metasurface reveals a clean EQ excitation. To the best of our knowledge, such clean quadrupole excitation free of coupled dipolar contents has not been previously demonstrated in the metasurface platform.^70–72^ By switching the adjoining sources to four in-plane magnetic dipoles, the same scheme yields a freeform metasurface supporting a clean magnetic quadrupole (MQ) resonance (Figures 3c). We anticipate that our adjoint-source formulation can extend the framework to other complex, nontrivial modes, such as toroidal^68,69,73,74^ and anapole^75,76^ resonances, which we leave for future work.

**Figure 3:**
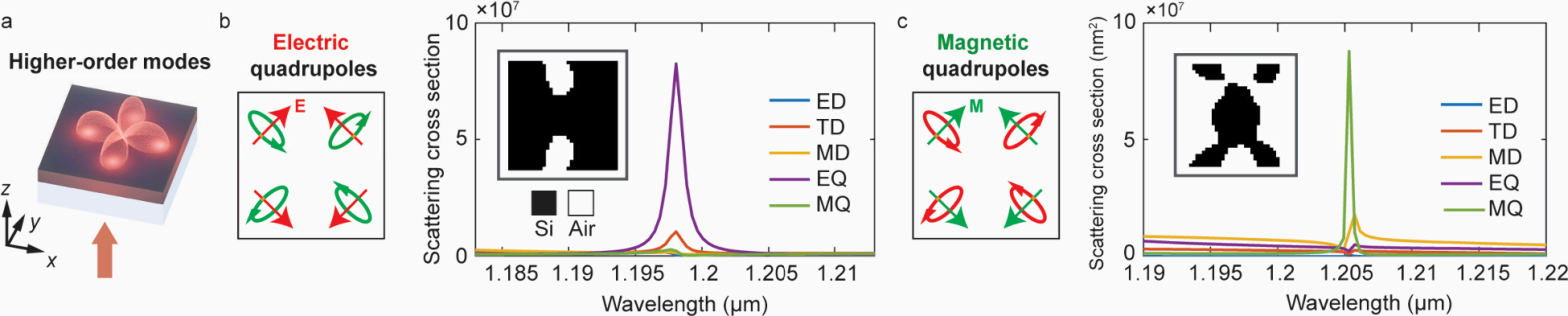
Higher-order mode engineering. (a) Schematic of the design setup for quadrupole modes. (b, c) Electric quadrupole (EQ) (b) and magnetic quadrupole (MQ) (c) designs by optimizing four in-plane oriented dipoles inside the unit cell. The multipolar decompositions of the scattering cross section show a dominant EQ/MQ resonance, confirming clean mode contents. The unit-cell periods are 720 nm in both cases.

Our computational optimization framework can be readily extended to multifunctional metasurfaces, including the multiplexed excitation of various Mie modes with distinct properties within a single unit cell. As a first demonstration, we design a multiwavelength nonlocal freeform metasurface that supports a pair of dipole resonances, each operating at distinct wavelengths and featuring distinct mode symmetries and resonant properties. The optimization setup is shown in the schematic in Figure 4a and shows the specification of two Mie-type modes in a periodic meta-array, an in-plane electric dipole (*p*_*x*_) and an out-of-plane magnetic dipole (*m*_*z*_) at the wavelengths of 1.3 and 1.365 μm, respectively. To co-optimize the two target modes, we define a composite FoM within the metasurface as the sum of the ED and MD mode intensities, *|* *E*_*x*_(λ_1_)*|* ^2^ + *|* *H*_*z*_(λ_2_)*|* ^2^. The optimization trajectory of the mode intensities (log scale) at the two target wavelengths is shown in Figure 4b, indicating a consistent increase in FoM over the course of optimization. The simulated transmission spectrum (Figure 4c) and multipolar decomposition (Figure 4d) confirm strong electric and magnetic resonances hosted within the metasurface (see more detailed modal analysis in Supplementary Section 7).

**Figure 4:**
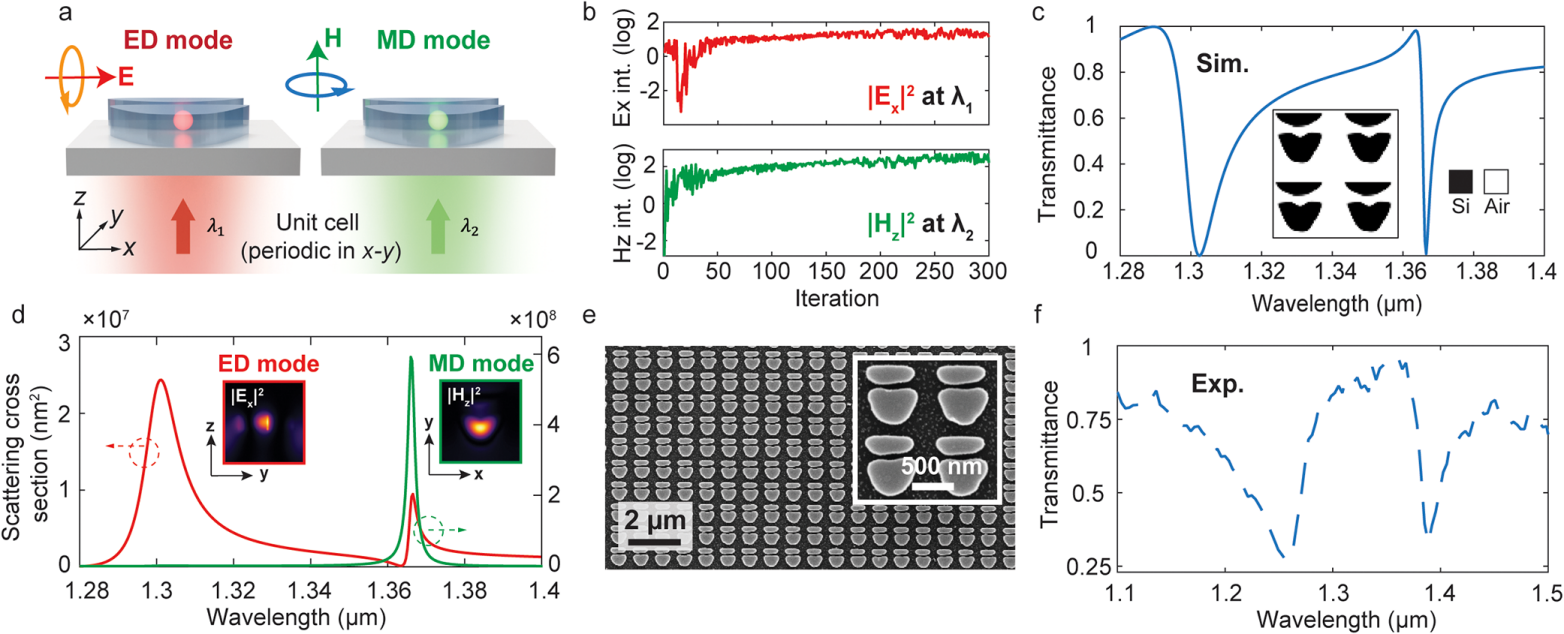
Multispectral freeform mode engineering. (a) Design implementation for a multiwavelength mode-engineered nonlocal metasurface. The unit-cell period is 870 nm. The device is designed to support an in-plane electric dipole (ED) resonance and an out-of-plane magnetic dipole (MD) mode at two different wavelengths. (b) Optimization trajectory showing the log-scale increase of electric and magnetic field intensities at the two target wavelengths. (c) Simulated transmission spectrum of the optimized structure. Inset: top view of the freeform device layout. (d) Multipolar decomposition of the near-fields. Insets: field profiles of the two target modes. (e) Scanning electron microscope image of the fabricated device. (f) Measured experimental transmission spectrum.

We experimentally validate the design by fabricating the optimized metasurface within a 150 nm-thick polycrystalline silicon film on a fused silica substrate. The metasurface patterns are defined using electron beam lithography and reactive ion etching. Figure 4e presents the top-view scanning electron microscopy (SEM) images of the meta-atoms, showing well-defined geometric features consistent with the design. The measured transmission spectrum (Figure 4f) confirms the presence of the two target dipole modes with broadened line widths due to slight off-normal incidence of the beam (see Supplementary Section 8).

As a second demonstration, we design a freeform metasurface that utilizes spectrally overlapped multimode resonances to produce planar chiral nonlocal responses,^53,77^ specifically spin-selective responses that are exclusively induced by circularly polarized light of a specific handedness (Figure 5a). Single-layer metamaterial systems supporting strong chiroptical responses generally require the electric and magnetic fields to be collinear and spatially overlapped within the dielectric medium.^78^ To achieve these criteria, we use multiobjective optimization to specify spectrally and spatially overlapping *E*_*z*_ and *H*_*z*_ dipole modes that support collinear electric and magnetic field components (Figure 5b). We specifically define the multivariate FoM to be *|* *E*_*z*_ *||* *H*_*z*_ *|* at a point within the metasurface to promote a balanced ED/MD response, and the FoM is specified to be maximized only when the metasurface is illuminated by left-hand polarized light (LCP) incidence.

**Figure 5:**
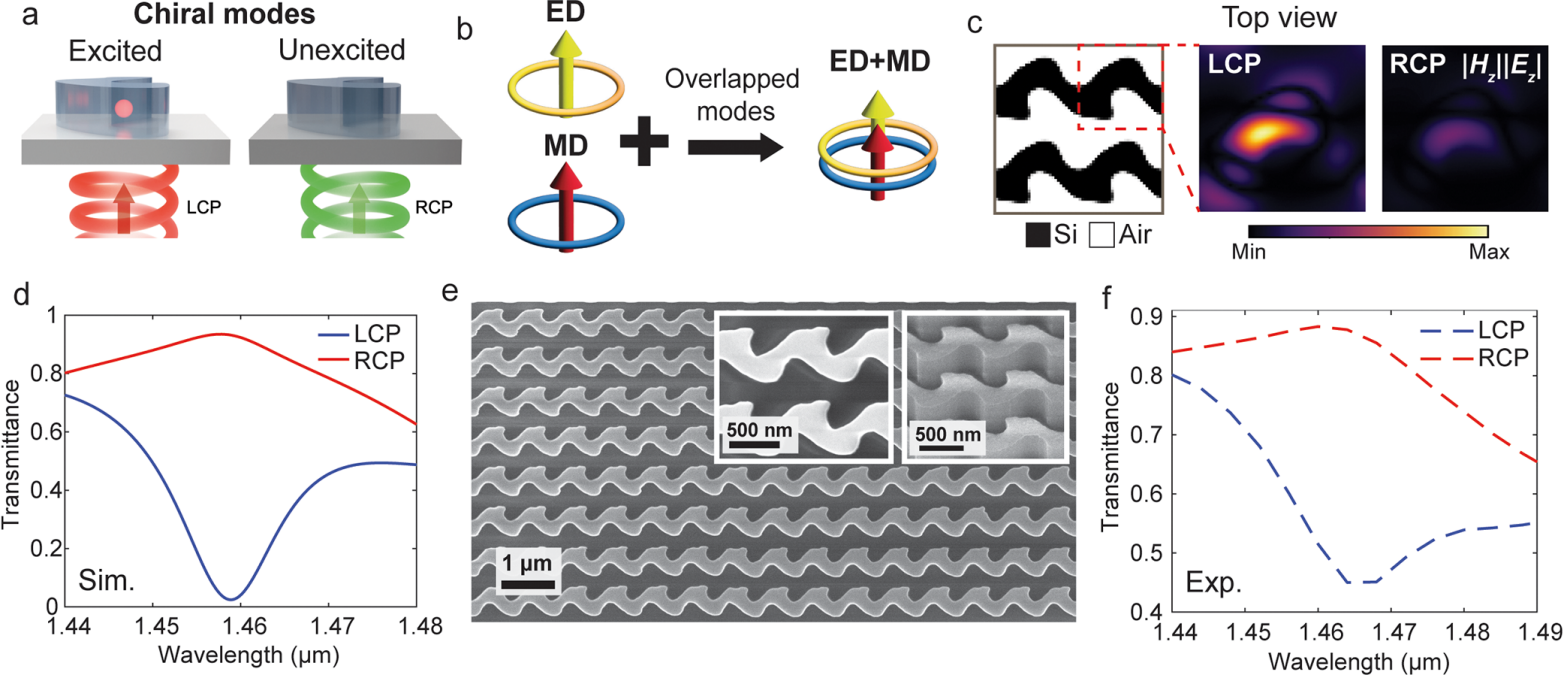
Multiresonant chiral mode engineering. (a) Design implementation showing that the chiral modes are excited exclusively under left-hand polarized light (LCP) incidence and remain unexcited for right-hand polarized light (RCP) incidence. (b) Multiresonant mode design concept involving a pair of spectrally overlapped electric dipole and magnetic dipole modes. (c) Top view of the optimized metasurface (left), with the corresponding *|* *E*_*z*_ *||* *H*_*z*_ *|* near-field distributions for LCP (center) and RCP (right) illumination. The unit-cell period is 750 nm. (d) Simulated transmission spectra for LCP and RCP incidence. (e) Scanning electron microscope images of the fabricated device. (f) Measured experimental transmission spectra for LCP and RCP incidence.

The metasurface is designed for a wavelength of 1460 nm and a period of 750 nm. The silicon layer thickness is specified to be 450 nm, and it is relatively thicker compared to prior demonstrations to break the radiation symmetry in the forward and backward directions. A top view of the optimized chiral metasurface is shown in Figure 5c (left), and the corresponding near-field |*E*_*z*_|*|* *H*_*z*_ *|* distributions under LCP (Figure 5c, middle) and RCP (Figure 5c, right) illumination show strongly selective Mie mode excitations. The simulated transmission spectra under LCP and RCP illumination, measured by the total transmitted power (Figure 5d), show a 94% circular dichroism (CD) response at the design wavelength (see additional modal and CP-conversion analyses in Supplementary Sections 9 and 10).

We experimentally validate the design by patterning and etching a 450 nm-thick silicon film on a fused silica substrate, and the top-view SEM image of the fabricated device is shown in Figure 5e. The left inset provides a close-up view of the nanoscale features, revealing well-defined curvilinear geometries with smooth, vertical sidewalls. The measured transmission spectra for the two CP illuminations (Figure 5f) show spectral line shapes that match well with the simulation. We attribute the reduced CD and broadened line width in the experimental device to fabrication imperfections and the slight off-normal incidence of the laser beam (see Supplementary Section 11). Further enhancements can be achieved by imposing more stringent feature size constraints to mitigate sensitivity to fabrication imperfections.^27,79–81^

In summary, in this work, we have introduced and demonstrated a near-field inverse design framework to realize freeform resonant metasurfaces through the explicit engineering of optical modes. Our approach accounts for and exploits the complex interplay between nanoscale freeform shapes and optical near-fields, which enables full customization of optical modes in both spatial and spectral domains through efficient exploration of the freeform design space. We anticipate many future extensions of this work. One is the extension of our concepts beyond single-layer media to multilayer^82–86^ and aperiodic^87^ nonlocal metasurfaces, which can lead to qualitatively new regimes of mode multiplexing capabilities. Another involves incorporating spatial perturbations into engineered nonlocality to achieve spatial and momentum light control.^35,88^ One other opportunity explores the utilization of faster electromagnetic solvers and optimizers,^89,90^ which may address current computational bottlenecks in throughput and speed. On the application front, we anticipate that our ability to customize optical near-fields has the potential to impact many application domains including molecular sensing, where conventional nonlocal metasurfaces are limited due to the location of hotspots within the metasurface nanostructures and where our platform can be used to define customized hotspots in near-field regions outside of the metasurface nanostructures. Multifunctional hotspot engineering also has applications in nonlinear optics,^8,9^ optomechanics,^91,92^ and quantum emission enhancement^7^ and photochemistry,^93,94^ where strong and tailored light–matter interactions are required.

## Supplementary Material



## Notes

Supplementary Sections 1–11: (1) coupled mode theory formulation; (2) air padding implementation; (3) hybrid optimization details; (4) angular dispersion of accidental BIC; (5) near-field analyses of accidental BIC metasurfaces; (6) additional freeform quasi-BIC designs; (7) multipolar decomposition of multiwavelength design; (8) multiwavelength design under off-normal incidence; (9) multipolar decomposition of chiral design; (10) circular polarization conversion analysis; (11) chiral metasurface under oblique incidence (PDF)

## References

[1] Tittl A. (2018). Imaging-based molecular barcoding with pixelated dielectric metasurfaces. Science.

[2] Khan S. A. (2022). Optical Sensing by Metamaterials
and Metasurfaces: From Physics to
Biomolecule Detection. Adv. Opt Mater..

[3] Wang X. (2024). Advances in information
processing and biological imaging using flat
optics. Nature Reviews Electrical Engineering.

[4] Noda S., Fujita M., Asano T. (2007). Spontaneous-emission
control by photonic
crystals and nanocavities. Nat. Photonics.

[5] Pelton M. (2015). Modified spontaneous
emission in nanophotonic structures. Nat. Photonics.

[6] Russell K. J., Liu T.-L., Cui S., Hu E. L. (2012). Large spontaneous
emission enhancement in plasmonic nanocavities. Nat. Photonics.

[7] Khoram E., Yu Z., Hassani Gangaraj S. A. (2024). Adjoint-Optimized
Large Dielectric
Metasurface for Enhanced Purcell Factor and Directional Photon Emission. ACS Omega.

[8] Koshelev K. (2020). Subwavelength dielectric resonators for nonlinear
nanophotonics. Science.

[9] Koshelev K. (2019). Nonlinear Metasurfaces
Governed by Bound States in the Continuum. ACS
Photonics.

[10] Hughes T. W., Minkov M., Williamson I. A. D., Fan S. (2018). Adjoint Method and
Inverse Design for Nonlinear Nanophotonic Devices. ACS Photonics.

[11] Almeida E., Shalem G., Prior Y. (2016). Subwavelength nonlinear
phase control
and anomalous phase matching in plasmonic metasurfaces. Nat. Commun..

[12] Yang K. Y. (2020). Inverse-designed non-reciprocal pulse router for chip-based LiDAR. Nat. Photonics.

[13] Kullock, R. ; Ochs, M. ; Grimm, P. ; Emmerling, M. ; Hecht, B. Electrically-driven Yagi-Uda antennas for light. Nat. Commun. 11, 115 (2020). 10.1038/s41467-019-14011-6 31913288 PMC6949256

[14] Babicheva V. E., Evlyukhin A. B. (2017). Resonant
lattice Kerker effect in metasurfaces with
electric and magnetic optical responses. Laser
Photon Rev..

[15] Fan Z. (2018). Perfect
diffraction with multiresonant bianisotropic metagratings. ACS Photonics.

[16] Alaee R. (2015). All-dielectric reciprocal bianisotropic nanoparticles. Phys. Rev. B.

[17] Pfeiffer C., Zhang C., Ray V., Guo L. J., Grbic A. (2014). High performance
bianisotropic metasurfaces: Asymmetric transmission of light. Phys. Rev. Lett..

[18] Pfeiffer C., Grbic A. (2014). Bianisotropic metasurfaces
for optimal polarization control: Analysis
and synthesis. Phys. Rev. Appl..

[19] Decker M. (2015). High-Efficiency Dielectric
Huygens’ Surfaces. Adv. Opt Mater..

[20] Pfeiffer C. (2014). Efficient light bending
with isotropic metamaterial Huygens’
surfaces. Nano Lett..

[21] Liu S. (2017). Huygens’ Metasurfaces
Enabled by Magnetic Dipole Resonance
Tuning in Split Dielectric Nanoresonators. Nano
Lett..

[22] Cai W., Chettiar U. K., Kildishev A. V., Shalaev V. M. (2007). Optical cloaking
with metamaterials. Nat. Photonics.

[23] Schurig D. (2006). Metamaterial electromagnetic
cloak at microwave frequencies. Science.

[24] Alù A., Engheta N. (2008). Multifrequency optical invisibility cloak with layered
plasmonic shells. Phys. Rev. Lett..

[25] Sell D., Yang J., Doshay S., Yang R., Fan J. A. (2017). Large-Angle,
Multifunctional Metagratings Based on Freeform Multimode Geometries. Nano Lett..

[26] Ra’di Y., Sounas D. L., Alu A. (2017). Meta-Gratings: Beyond
the Limits
of Graded Metasurfaces for Wavefront Control. Phys. Rev. Lett..

[27] Zhou Y., Mao C., Gershnabel E., Chen M., Fan J. A. (2024). Large-Area, High-Numerical-Aperture,
Freeform Metasurfaces. Laser Photon Rev..

[28] Hsu, C. W. ; Zhen, B. ; Stone, A. D. ; Joannopoulos, J. D. ; Soljacic, M. Bound states in the continuum. Nature Reviews Materials. 2016, 1, 10.1038/natrevmats.2016.48.

[29] Koshelev K., Bogdanov A., Kivshar Y. (2019). Meta-optics and bound
states in the
continuum. Sci. Bull. (Beijing).

[30] Overvig A., Alù A. (2021). Wavefront-selective
Fano resonant metasurfaces. Advanced Photonics.

[31] Overvig A. C., Malek S. C., Carter M. J., Shrestha S., Yu N. (2020). Selection
rules for quasibound states in the continuum. Phys. Rev. B.

[32] Markowitz M. (2022). Tailored resonant waveguide gratings for augmented
reality. Opt Express.

[33] Zhou Y., Guo S., Overvig A. C., Alù A. (2023). Multiresonant Nonlocal Metasurfaces. Nano Lett..

[34] Overvig A. C., Malek S. C., Yu N. (2020). Multifunctional Nonlocal
Metasurfaces. Phys. Rev. Lett..

[35] Malek, S. C. ; Overvig, A. C. ; Alù, A. ; Yu, N. Multifunctional resonant wavefront-shaping meta-optics based on multilayer and multi-perturbation nonlocal metasurfaces. Light Sci. Appl. 11, 246 (2022). 10.1038/s41377-022-00905-6 35922413 PMC9349264

[36] Overvig A., Alù A. (2022). Diffractive Nonlocal Metasurfaces. Laser Photon Rev..

[37] Koshelev K., Lepeshov S., Liu M., Bogdanov A., Kivshar Y. (2018). Asymmetric
Metasurfaces with High- Q Resonances Governed by Bound States in the
Continuum. Phys. Rev. Lett..

[38] Yesilkoy F. (2019). Ultrasensitive hyperspectral
imaging and biodetection enabled by
dielectric metasurfaces. Nat. Photonics.

[39] Ha S. T. (2018). Directional lasing in
resonant semiconductor nanoantenna arrays. Nat.
Nanotechnol.

[40] Zhou Y., Zheng H., Kravchenko I. I., Valentine J. (2020). Flat optics
for image differentiation. Nat. Photonics.

[41] Overvig A. C. (2023). Zone-folded quasi-bound
state metasurfaces with customized, symmetry-protected
energy-momentum relations. ACS Photonics.

[42] Niederberger A. C. R., Fattal D. A., Gauger N. R., Fan S., Beausoleil R. G. (2014). Sensitivity
analysis and optimization of sub-wavelength optical gratings using
adjoints. Opt Express.

[43] Lalau-Keraly C. M., Bhargava S., Miller O. D., Yablonovitch E. (2013). Adjoint shape
optimization applied to electromagnetic design. Opt Express.

[44] Zhou M. (2021). Inverse design of metasurfaces based on coupled-mode theory and adjoint
optimization. ACS Photonics.

[45] Zhang D., Liu Z., Yang X., Xiao J. J. (2022). Inverse Design of Multifunctional
Metasurface Based on Multipole Decomposition and the Adjoint Method. ACS Photonics.

[46] Roques-Carmes C. (2022). Toward 3D-Printed Inverse-Designed Metaoptics. ACS Photonics.

[47] Roberts G. (2023). 3D-patterned inverse-designed mid-infrared metaoptics. Nat. Commun..

[48] Pestourie R. (2018). Inverse design of large-area
metasurfaces. Opt. Express.

[49] Chen M. (2024). Validation and characterization
of algorithms and software for photonics
inverse design. Journal of the Optical Society
of America B.

[50] Jiang J., Fan J. A. (2019). Global Optimization
of Dielectric Metasurfaces Using
a Physics-Driven Neural Network. Nano Lett..

[51] Suh W., Wang Z., Fan S. (2004). Temporal coupled-mode
theory and
the presence of non-orthogonal modes in lossless multimode cavities. IEEE J. Quantum Electron..

[52] Fan S., Suh W., Joannopoulos J. D. (2003). Temporal
coupled-mode theory for
the Fano resonance in optical resonators. JOSA
A.

[53] Shi T. (2022). Planar chiral metasurfaces
with maximal and tunable chiroptical response
driven by bound states in the continuum. Nat.
Commun..

[54] Bahmani S., Evlyukhin A. B., Hassan E., Calà Lesina A. (2025). Topology optimization
of optical nanoantennas with desired multipoles. Opt Express.

[55] Koshelev K., Kivshar Y. (2021). Dielectric Resonant
Metaphotonics. ACS Photonics.

[56] Dai T. (2025). Shaping freeform nanophotonic
devices with geometric neural parameterization. NPJ. Comput. Mater..

[57] Hinamoto T., Fujii M. (2021). MENP: an open-source
MATLAB implementation of multipole expansion
for nanophotonics. OSA Contin.

[58] Yoon J. W., Song S. H., Magnusson R. (2015). Critical field
enhancement of asymptotic
optical bound states in the continuum. Sci.
Rep.

[59] Mobini E., Alaee R., Boyd R. W., Dolgaleva K. (2021). Giant asymmetric
second-harmonic generation in bianisotropic metasurfaces based on
bound states in the continuum. ACS Photonics.

[60] Evlyukhin A. B. (2021). Polarization switching
between electric and magnetic quasi-trapped
modes in bianisotropic all-dielectric metasurfaces. Laser Photon Rev..

[61] Kang M., Zhang S., Xiao M., Xu H. (2021). Merging bound states
in the continuum at off-high symmetry points. Phys. Rev. Lett..

[62] Sidorenko M. S. (2021). Observation of an accidental
bound state in the continuum in a chain
of dielectric disks. Phys. Rev. Appl..

[63] Mizuno Y., Tsutsui K., Tohyama T., Maekawa S. (2000). Nonlinear optical response
and spin-charge separation in one-dimensional Mott insulators. Phys. Rev. B.

[64] McMahon J. M., Li S., Ausman L. K., Schatz G. C. (2012). Modeling
the effect of small gaps
in surface-enhanced Raman spectroscopy. J. Phys.
Chem. C.

[65] Aikens C.
M., Madison L. R., Schatz G. C. (2013). The effect of field gradient on SERS. Nat. Photonics.

[66] Chen Y. (2022). Dipole-forbidden transitions induced by the gradient
of optical near
fields. Phys. Rev. A (Coll Park).

[67] Rusak E. (2019). Enhancement of and interference
among higher order multipole transitions
in molecules near a plasmonic nanoantenna. Nat.
Commun..

[68] Savinov V., Fedotov V. A., Zheludev N. I. (2014). Toroidal
dipolar excitation and macroscopic
electromagnetic properties of metamaterials. Phys. Rev. B.

[69] Papasimakis N., Fedotov V. A., Savinov V., Raybould T. A., Zheludev N. I. (2016). Electromagnetic
toroidal excitations in matter and free space. Nat. Mater..

[70] Liu C. (2020). Beyond dipole excitation:
the performance of quadrupole-based Huygens’
metasurface. Opt. Lett..

[71] Babicheva V. E., Evlyukhin A. B. (2018). Metasurfaces
with Electric Quadrupole and Magnetic
Dipole Resonant Coupling. ACS Photonics.

[72] Shevchenko A., Kivijärvi V., Grahn P., Kaivola M., Lindfors K. (2015). Bifacial metasurface
with quadrupole optical response. Phys. Rev.
Appl..

[73] Kaelberer T., Fedotov V. A., Papasimakis N., Tsai D. P., Zheludev N. I. (2010). Toroidal
dipolar response in a metamaterial. Science.

[74] Basharin A. A. (2015). Dielectric Metamaterials with Toroidal Dipolar Response. Phys. Rev. X.

[75] Wu P. C. (2018). Optical anapole metamaterial. ACS Nano.

[76] Tripathi A. (2021). Lasing Action from Anapole Metasurfaces. Nano
Lett..

[77] Overvig, A. ; Yu, N. ; Alù, A. Chiral Quasi-Bound States in the Continuum. Phys. Rev. Lett. 126, (2021). 10.1103/PhysRevLett.126.073001 33666456

[78] Zhu A. Y. (2018). Giant intrinsic chiro-optical activity in planar dielectric
nanostructures. Light Sci. Appl..

[79] Zhou Y., Shao Y., Mao C., Fan J. A. (2024). Inverse-designed
metasurfaces with facile fabrication parameters. Journal of Optics.

[80] Wang E. W., Sell D., Phan T., Fan J. A. (2019). Robust
design of
topology-optimized metasurfaces. Opt Mater.
Express.

[81] Wang F., Jensen J. S., Sigmund O. (2011). Robust topology
optimization of photonic
crystal waveguides with tailored dispersion properties. JOSA B.

[82] Zhou Y. (2018). Multilayer Noninteracting Dielectric Metasurfaces for Multiwavelength
Metaoptics. Nano Lett..

[83] Lin Z., Groever B., Capasso F., Rodriguez A. W., Lončar M. (2018). Topology-Optimized Multilayered Metaoptics. Phys. Rev. Appl..

[84] Zhou Y. (2019). Multifunctional metaoptics
based on bilayer metasurfaces. Light Sci. Appl..

[85] Avayu O., Almeida E., Prior Y., Ellenbogen T. (2017). Composite
functional metasurfaces for multispectral achromatic optics. Nat. Commun..

[86] Zheng H. (2022). Compound Meta-Optics for Complete and Loss-Less Field Control. ACS Nano.

[87] Li C., Liu C., Peters C., Yu H., Maier S. A., Forbes A., Ren H. (2025). Disorder-enabled Synthetic Metasurfaces. arXiv
(physics.optics).

[88] Overvig, A. ; Mann, S. A. ; Alù, A. Spatio-temporal coupled mode theory for nonlocal metasurfaces. Light Sci. Appl. 13, 28 (2024). 10.1038/s41377-023-01350-9 38263149 PMC11251021

[89] Augenstein, Y. ; Repän, T. ; Rockstuhl, C. Neural Operator-Based Surrogate Solver for Free-Form. Electromagnetic Inverse Design. ACS Photonics 2023, 10, 1547 10.1021/acsphotonics.3c00156.

[90] Chen M. (2022). High speed simulation
and freeform optimization of nanophotonic devices
with physics-augmented deep learning. ACS Photonics.

[91] Aspelmeyer M., Kippenberg T. J., Marquardt F. (2014). Cavity optomechanics. Rev. Mod.
Phys..

[92] Barzanjeh S. (2022). Optomechanics for quantum technologies. Nat.
Phys..

[93] Yuen-Zhou J., Menon V. M. (2019). Polariton chemistry: Thinking inside the (photon) box. Proc. Natl. Acad. Sci. U. S. A..

[94] Wu Y., Yang W., Fan Y., Song Q., Xiao S. (2019). TiO2 metasurfaces:
From visible planar photonics to photochemistry. Sci. Adv..

