# Supplementary material for: Freeform Mode-Engineered Metasurfaces
Source: Nano Lett. 2026 Mar 10;26(11):3752–9. doi: 10.1021/acs.nanolett.5c06075 (PMC13022851; doi:10.1021/acs.nanolett.5c06075)
Supplement: Supplementary file 1 [file nl5c06075_si_001.pdf]

Supplementary Information for:

Freeform Mode-Engineered Metasurfaces

Zhongjun Jiang[1], Tianxiang Dai[2], Shuwei Guo[3], Soyaib H. Sohag[1], Yixuan Shao[2], Chenkai Mao[2], Andrea Alù[3,4*] and Jonathan A. Fan[2*], You Zhou[1*]

[1]Department of Physics and Optical Science, University of North Carolina, Charlotte, 28223, USA

[2]Department of Electrical Engineering, Stanford University, Stanford, California, 94305, USA

[3]Photonics Initiative, Advanced Science Research Center, City University of New York, New York, NY, 10031, USA

[4]Physics Program, Graduate Center, City University of New York, New York, NY, 10016, USA

[*]Email: yzhou33@charlotte.edu, jonfan@stanford.edu, aalu@gc.cuny.edu

Section 1: Coupled mode theory

In the Temporal coupled mode theory (TCMT) formulation (depicted in Figure S1), the coupling parameter $\mathbf{K}$ describes the coupling between the desired modes and the incident free-space excitation. The near-field is primarily determined by the modal properties encoded in the mode-amplitude vector $\mathbf{a}$, with the resonance frequencies and decay rates described by $\mathbf{\Omega}$ and $\mathbf{\Gamma}$, respectively. The far-field response is governed by two pathways: (i) the direct scattering channel $\mathbf{C}\,|s_+\rangle$, where $\mathbf{C}$ is the direct coupling matrix between input and output channels, and (ii) the resonant pathway through the modes, $\mathbf{Da}$, where $\mathbf{D}$ describes coupling from the modes to the output channels $|s_-\rangle$. The mode-amplitude vector $\mathbf{a}$ holds the essential modal properties in the near-field and implicitly transforms the incoming wave into an outgoing wave (that can be detected in the far-field) based on the TCMT framework.

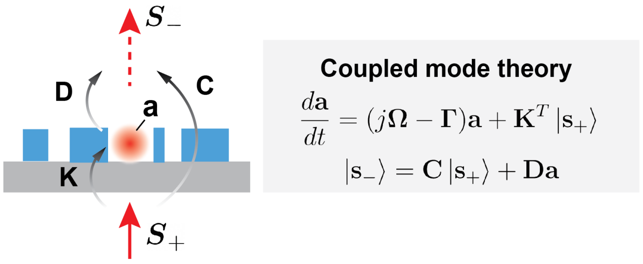

Figure S1 | Coupled mode theory.

Section 2: Air padding implementation

During the optimization, the unit cell boundary is laterally padded with an air buffer to isolate the meta-atom structures (Figure S2a). This padding helps suppress unwanted higher-order or guided wave modes that commonly arise in connected Si patterns. Our initial optimization was performed without such air padding, which produced continuous Si connections between adjacent units (Figure S2b). The multipolar decomposition reveals an undesired magnetic quadrupole overlayed with the target m_z mode, which led us to add such air padding for mode isolation. While complete suppression of undesired modes cannot be guaranteed, this empirical constraint consistently yields the clean mode contents shown in Figs. 2–4.

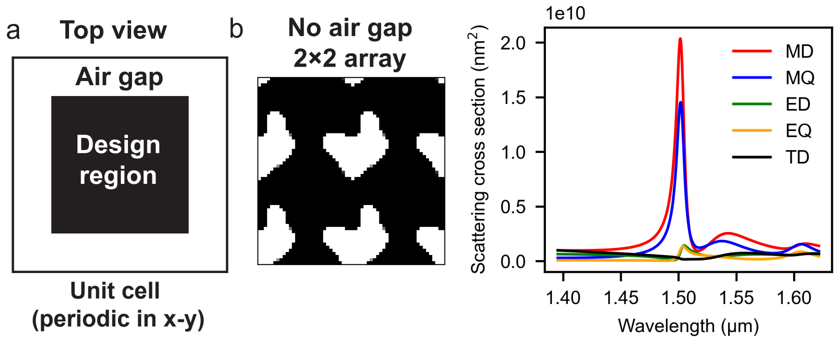

Figure S2 | (a) Schematic of an air buffer layer around the unit cell boundary. (b) m_z mode (MD) design without air padding. The multipolar decomposition reveals excitation of an undesired magnetic quadrupole (MQ). Abbreviations: MD (magnetic dipole), MQ (magnetic quadrupole), ED (electric dipole), EQ (electric quadrupole), TD (toroidal dipole).

Section 3: Hybrid optimization details

The optimization framework employed in this work utilizes a hybrid strategy combining density-based topology optimization (TO) with a subsequent high-resolution boundary optimization stage enabled by Neuroshaper. This two-stage approach, illustrated in Figure S3, facilitates efficient exploration of the design space (via TO) followed by precise refinement of geometric features, which is critical for engineering high-Q resonances.

1) The adjoint variables method (AVM) based topology optimization (Figure S3, yellow loop) starts with an initial guess of weighted topology density profile ρ ($\rho \in [0,1]$, where 0 and 1 represent surrounding and hosting materials, respectively). For this study, we choose silicon as the high-index material with $n_{Si} = 3.5$. To meet the requirement of device fabrication, the design field is filtered and projected (denoted by $\bar{\rho}$). Then the variables $\bar{\rho}$ are sent to a well-established open-

source electromagnetic solver Meep. The forward fields $\mathbf{E}_{\text{forward}}$ along with the figure of merit are subsequently calculated, with the excitation of a far-field source (e.g., a (quasi) plane wave, which is compatible with the experimental excitations). The adjoint fields $\mathbf{E}_{\text{adjoint}}$ are determined from a similarly configured simulation (i.e., the adjoint simulation) but the excitation source is replaced by near-field adjoint dipole sources $(\mathbf{P}, \mathbf{M})$, which are related to the pre-defined figure of merit function $F(\mathbf{E}, \mathbf{H})$, i.e., $\mathbf{P} = \partial F/\partial \mathbf{E}$ and $\mathbf{M} = -(1/\mu_0)(\partial F/\partial \mathbf{H})$. Combining the forward and adjoint fields, the variation ∂F can be immediately calculated as $2\text{Re}(\mathbf{E}_{\text{forward}} \cdot \mathbf{E}_{\text{adjoint}})$. The gradient with respect to the design variables ρ , hence, is given by $\frac{\partial F}{\partial \bar{\rho}} \cdot \frac{\partial \bar{\rho}}{\partial \rho}$. The geometery is updated accrodingly with the gradient and the iteration continues till the stop condition (e.g., the iteration number is reached or a 99% binarized design is obtained) is met. It is at this point a low-resolution design *f*(N) is obtained, which is ready for the next stage optimization for Q-factors control.

2) In the second stage (Figure S3, green loop), we employ Neuroshaper for boundary optimization. Neuroshaper utilizes a neural level set $F_\theta(x, y)$ (composed of a Lookup Table and MLP) to provide an analytic, high-resolution representation of the geometry.

A key aspect of this methodology is the initialization of the Neuroshaper representation directly from the results of the topology optimization. The low-resolution design *f*(N) is used to initialize the neural level set parameters (θ). This initialization is performed using a two-objective fitting process:

1. Boundary Similarity (BCE loss): Ensuring the neural representation closely matches the boundary of the topology-optimized design.
2. Geometric Constraints: Imposing feature size and curvature constraints (Penalty $L_2'(M)$).

This process generates a faithful, high-resolution design $M(\theta)$ that adheres to fabrication constraints while preserving the optimized topology from Stage 1.

With this high-resolution representation, boundary optimization proceeds using AVM. The gradients of the objective function $L_1(M)$ and the constraints $L_2'(M)$ are estimated with respect to the neural parameters θ. By iteratively updating θ, we can precisely modify the high-resolution geometric features. This fine-grained control allows us to systematically tailor the Q-factor of the

resonance. Specifically, the optimization adjusts the geometry to modify the coupling pathways between the target mode and free-space radiation, thereby enhancing or decreasing the target H_z field values, which directly controls the Q-factor.

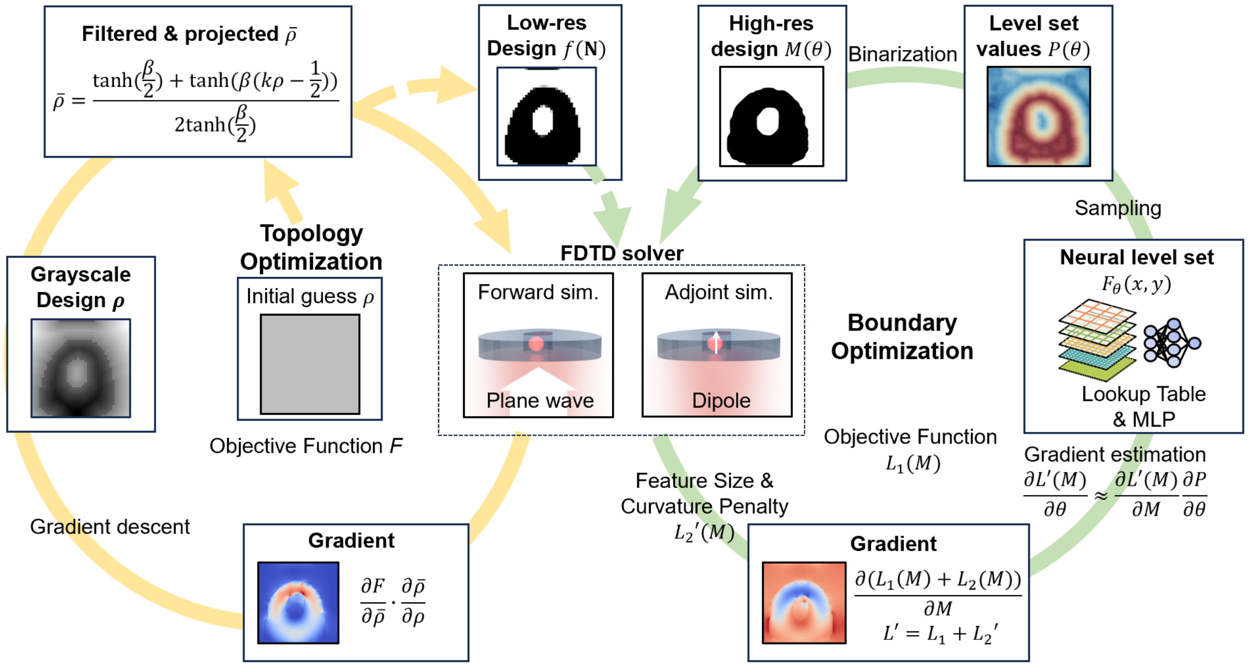

Figure S3 | Workflow of the hybrid optimization strategy combining Topology Optimization and Neuroshaper-enabled Boundary Optimization. The process involves two stages. The Topology Optimization stage (yellow loop) uses AVM on a grayscale design (ρ) to identify a promising topology $f(\mathbf{N})$. The Boundary Optimization stage (green loop) utilizes Neuroshaper (Neural level set $F_\theta(x, y)$). Neuroshaper is initialized from the TO results using a two-objective fitting (boundary similarity and geometric constraints) to generate a high-resolution design $M(\theta)$. This representation is then fine-tuned to control the Q-factor by optimizing the near-field response (L_1) while enforcing feature size and curvature penalties (L_2').

Section 4: Angular dispersion of normal-incidence and off-normal quasi-BIC designs

We compare the angular dispersion of quasi-BIC modes designed at normal incidence with those optimized individually for off-normal incidence. As shown in Figure S4a, the baseline device optimized only at normal incidence (left) shows strong angle-dependent shifts and Q degradation. We further re-optimized the same starting design at different angles (Figure S4b) and yields robust accidental BIC whose resonance wavelengths and Q-factors remain essentially constant over a wide range of angles.

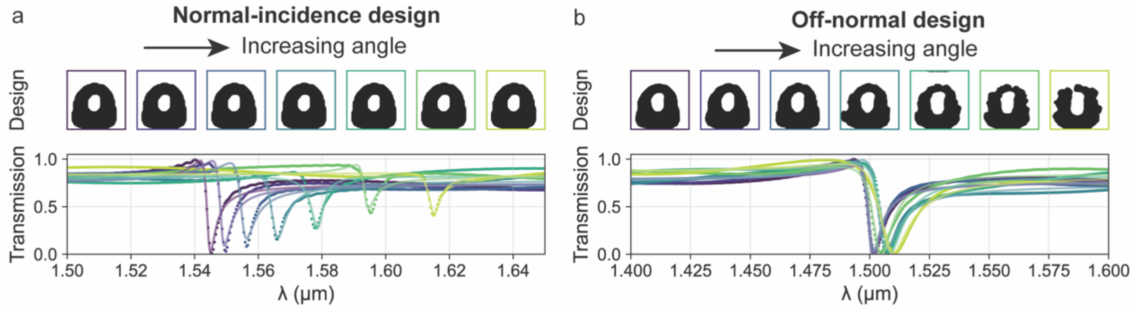

Figure S4 | Permittivity distributions of the meta-atoms and their corresponding transmission spectra for normal-incidence (a) and off-normal (b) designs.

Section 5: Near-field analyses of accidental BIC metasurfaces

Regarding the nature of the accidental BIC, it originates from parameter-tuned cancellation of tangential vector fields inside the nanostructures**,** which suppresses the in-plane dipolar components essential for free-space coupling. As shown in Fig. S5a, we map the vectorial near fields along a vertical cut through the center of the 'donut' design along with two additional designs (see Section 6 for details), revealing spatially varying field amplitudes and orientations. Summing the tangential field vectors over the unit cell yields an overall cancellation, $\sum E_{||}(x,y) \approx 0$. This carefully balanced field cancellation is achieved purely through precise structural engineering rather than global asymmetry. Another distinguishing feature of our accidental BIC mode is that the local vector-field distribution can appear irregular despite the global cancellation, in contrast to symmetry-protected schemes that typically exhibit more symmetric profiles (Fig. S5b).

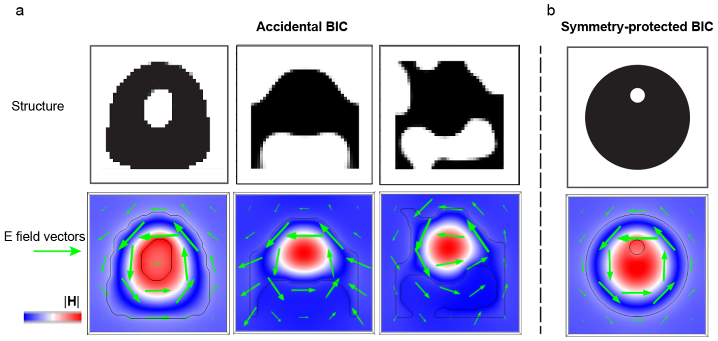

Figure S5 | Magnetic field and vectorial **E** field distributions of m_z mode resonant metasurface designs for the accidental BIC (a) and the symmetry-protected BIC (b).

Section 6: Additional accidental quasi-BIC designs

Our gradient-based optimization is non-convex and is highly sensitive to initial geometries. The asymmetric "donut" structure shown in Figure 2 is one local optimum obtained from a uniform-

density start (permittivity weight 0.2 between air and Si). While our asymmetric donut appears to be similar to those previously studied symmetry-protected BIC designs[1–3], our structure ultimately utilizes distinct physics and is distinctive in the following ways: (i) it utilizes an accidental BIC mechanism, which is distinctive from the symmetry protected BIC mechanism, and (ii) the similarities allows us to map the transition between accident and symmetry protected BIC schemes. Ultimately, beyond this example, our approach enables exploration of a significantly larger nonlocal design space beyond conventional templates. As shown in Figure S6, we optimized the same BIC mode from two random starts and obtained two physically non-intuitive geometries with the same modal content.

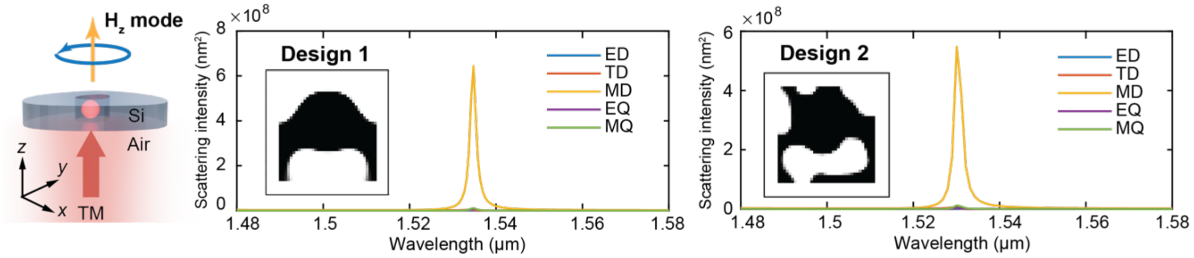

Figure S6 | Accidental quasi-BIC mode supported by diverse freeform geometries. The same quasi-BIC mode is achieved from two different starting points.

Section 7: Multipolar decomposition of multiwavelength nonlocal design

We perform multipolar decomposition of the scattering cross section for the multiwavelength nonlocal metasurface, which is designed to support an in-plane electric dipole (p_x) at 1.3 μm and an out-of-plane magnetic dipole (m_z) at 1.365 μm. We analyze the directional components of electric and magnetic dipolar terms in Cartesian coordinates. As shown in Figure S7, the scattering is dominated by the p_x component in the electric mode (left) and the m_z component in the magnetic mode (right), which are consistent with our design objectives.

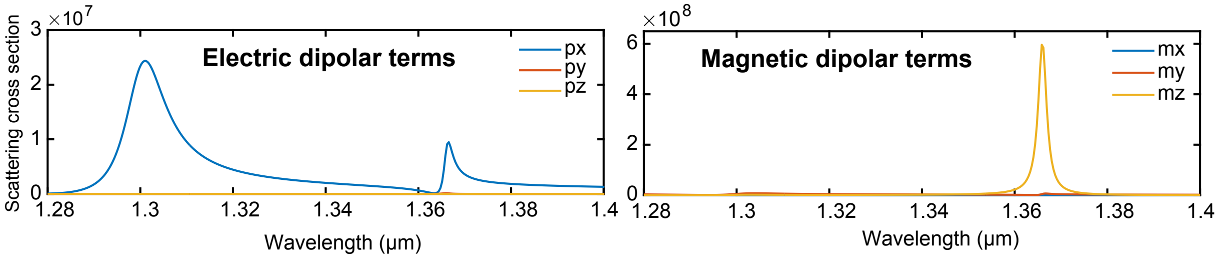

Figure S7 | Scattering properties of the multiwavelength nonlocal metasurface. (Left) Multipolar decomposition showing the Cartesian components of the electric dipolar response. (Right) Corresponding decomposition of the magnetic dipolar components.

Section 8: Multiwavelength resonant design under off-normal incidences

The designs in Figs. 4 are optimized under plane-wave normal incidence. In experiment, the illumination is a Gaussian beam with a finite divergence (~ a few degrees), which could drastically alter the Q factors since the nonlocal mode excitation is highly sensitive to momentum-matching conditions. To validate this, we calculated the transmission spectra for both designs under normal (θ = 0°) and oblique (θ = 5°) incidence (Fig. S8) and observed noticeable angularly dependent linewidth broadening.

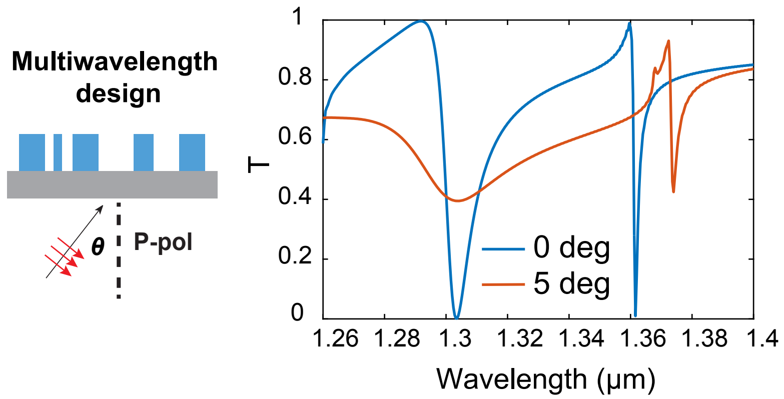

Figure S8 | Angle dependence of multiwavelength nonlocal metasurfaces under normal (θ = 0°) and oblique (θ = 5°) incidence.

Section 9: Multipolar decomposition of dual-mode chiral design

We perform multipole decomposition of the designed chiral metasurface that involves the design of spatially and spectrally overlapped E_z and H_z dipole modes under left-hand circularly polarized (LCP) excitation (see Figure 5 in the main text). The total scattering cross section was decomposed into electric dipole (ED), magnetic dipole (MD), and higher-order quadrupole terms (electric quadrupole (EQ), magnetic quadrupole (MQ)). The decomposition shown in Figure S9a reveals scattering predominantly governed by the combined ED and MD contributions. Figure S9b further presents the directional components of the dipolar terms in Cartesian coordinates (*x, y, z*), showing dominant contributions from the *z*-directed components. We specifically plot the z-oriented terms, because the chiral design target is two vertically oriented dipole modes ED_z/MD_z; their product serves as an axis-selective indicator of concurrent ED/MD excitation.

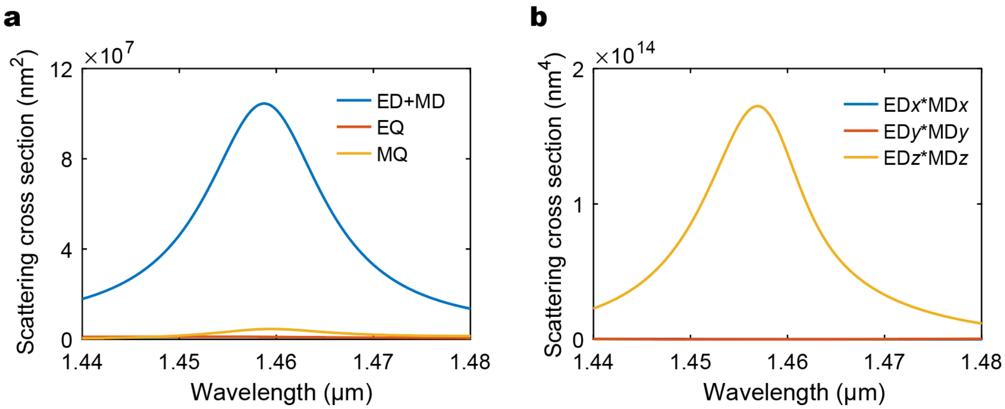

Figure S9 | Scattering properties of the dual-mode chiral metasurfaces. Multipole expansion (**a**) and the Cartesian components (**b**) of electric and magnetic dipolar terms.

Section 10: Circular polarization conversion of dual-mode chiral metasurface

The large circular dichroism (CD) in our dual-mode chiral design (Figure 5 in the main text) originates from spin-selective conversion of circularly polarized input (Figure S10a). The simulated co- and cross-polarized transmission spectra are shown in Figure S10b, revealing strong polarization conversion that occurs exclusively for RCP incidence.

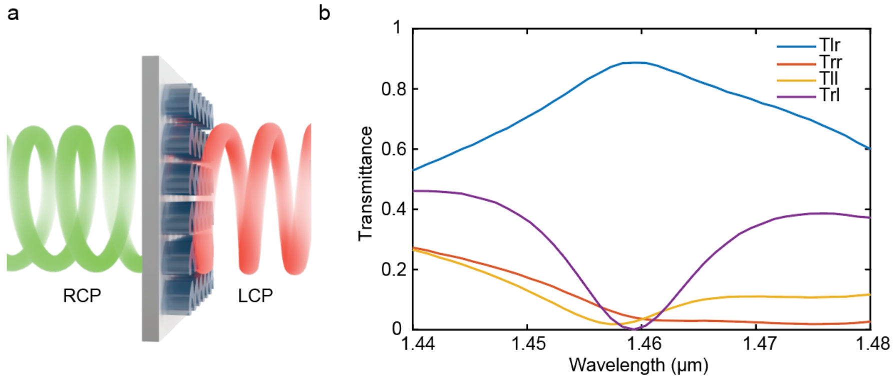

Figure S10 | Co- and cross-polarization transmission spectra of dual-mode chiral metasurface.

Section 11: Dual-mode chiral metasurface under oblique incidence

The reduced CD observed in the experimental chiral device can be primarily attributed to slight deviations from normal incidence. The simulated transmission spectra for LCP and RCP under normal (θ = 0°) and oblique (θ = 5°) incidence are shown in Figure S11b (left and right, respectively). The corresponding CD spectra is plotted in Figure S11c, where the CD value decreases from 95% at normal incidence to 50% at 5° incidence. In our experimental setup, the

illumination beam has a Gaussian profile with a finite divergence angle of a few degrees, which contributes to the reduced CD compared to the ideal normal incidence case.

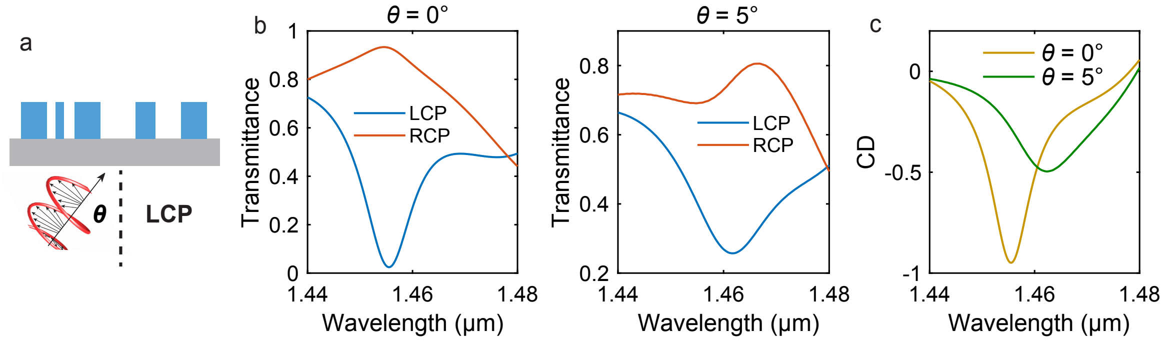

Figure S11 | Angle dependence of transmission spectra. (a) Transmission spectra under oblique incident angles. (b) Transmission spectra for LCP and RCP under normal (θ = 0°) and oblique (θ = 5°) incidence. (c) CD spectra under normal (θ = 0°) and oblique (θ = 5°) incidence.

References

1. Koshelev, K., Lepeshov, S., Liu, M., Bogdanov, A. & Kivshar, Y. Asymmetric Metasurfaces with High- Q Resonances Governed by Bound States in the Continuum. *Phys Rev Lett* **121**, 193903 (2018).

2. Mobini, E., Alaee, R., Boyd, R. W. & Dolgaleva, K. Giant asymmetric second-harmonic generation in bianisotropic metasurfaces based on bound states in the continuum. *ACS Photonics* **8**, 3234–3240 (2021).

3. Evlyukhin, A. B. *et al.* Polarization switching between electric and magnetic quasi-trapped modes in bianisotropic all-dielectric metasurfaces. *Laser Photon Rev* **15**, 2100206 (2021).
